# Proteomic and transcriptomic analyses of *Cutibacterium acnes* biofilms and planktonic cultures in presence of epinephrine

**DOI:** 10.3934/microbiol.2024019

**Published:** 2024-05-27

**Authors:** AV Gannesen, MI Schelkunov, RH Ziganshin, MA Ovcharova, MV Sukhacheva, NE Makarova, SV Mart'yanov, NA Loginova, AM Mosolova, EV Diuvenji, ED Nevolina, VK Plakunov

**Affiliations:** 1 Federal Research Centre “Fundamentals of Biotechnology” of Russian Academy of Sciences, Moscow 119071, Russia; 2 Skolkovo Institute of Science and Technology, Moscow 121205, Russia; 3 Institute for Information Transmission Problems of Russian Academy of Sciences, Moscow 127051, Russia; 4 Institute of Bioorganic Chemistry, Russian Academy of Sciences, Moscow 117997, Russia; 5 Russian Biotechnological University, Moscow 125080, Russia

**Keywords:** biofilms, *Cutibacterium acnes*, microbial endocrinology, proteomics, transcriptomics, RNA-Seq, qRT-PCR, epinephrine, hormones, skin microbiota

## Abstract

Transcriptomic and proteomic analysis were performed on 72 h biofilms of the acneic strain *Cutibacterium acnes* and planktonic cultures in the presence of epinephrine. Epinephrine predominantly downregulated genes associated with various transporter proteins. No correlation was found between proteomic and transcriptomic profiles. In control samples, the expression of 51 proteins differed between planktonic cultures and biofilms. Addition of 5 nM epinephrine reduced this number, and in the presence of 5 µM epinephrine, the difference in proteomic profiles between planktonic cultures and biofilms disappeared. According to the proteomic profiling, epinephrine itself was more effective in the case of *C. acnes* biofilms and potentially affected the tricarboxylic acid cycle (as well as alpha-ketoglutarate decarboxylase Kgd), biotin synthesis, cell division, and transport of different compounds in *C. acnes* cells. These findings are consistent with recent research on *Micrococcus luteus*, suggesting that the effects of epinephrine on actinobacteria may be universal.

## 1. Introduction

In microbial endocrinology, neurohormones and steroids were among the first hormone classes to be systematically studied as bacterial effectors [1],[2]. Epinephrine has been shown to have effects on some model bacteria, such as *Escherichia coli* [1]. Catecholamines increased the minimal inhibitory concentrations of antibiotics in *E. coli* and enhanced virulence [3]. Furthermore, the involvement of catecholamines, and epinephrine in particular, in bacterial two-component signaling systems, such as Qse in *E. coli* [4],[5], suggests a closer evolutionary relationship between humans and human microbiota. This hypothesis is further supported by the chemoattractive properties of catecholamines for *E. coli* [6]. In other bacteria, such as for example *Aeromonas hydrophila*, catecholamines cause genome-wide changes in gene expression and proteomic profiles [7]. In *Enterococcus faecalis*, epinephrine and norepinephrine have been reported to modulate the bacterial ability to adhere to eukaryotic tissues [8]. *Pseudomonas aeruginosa* responds to catecholamines by enhancing sensitivity to phages, increasing adhesion, altering motility (increasing swarming and modulating twitching), enhancing virulence, and stimulating biofilm formation [9]. In *Burkholderia pseudomallei*, epinephrine increased motility, flagella synthesis, and possibly iron uptake [10]; the latter was also reported for other species [11],[12]. These few selected examples establish epinephrine as a bacterial effector; for a recent review see Boukerb et al. [13].

The skin microbiota represents a particular challenge for the study of hormonal effects because it inhabits microenvironments where the diffusion of various compounds is poorly investigated, and thus the concentration of hormones remains unclear (despite some data on total hormone content in tissues [14]–[16]). Thus, the effects of catecholamines on human skin microbiota are still less investigated compared to the model gut microorganisms. Nevertheless, accumulated data suggest the universality of epinephrine effects on the microbiota. In *Staphylococcus aureus*, epinephrine promoted survival in the presence of lidocaine as a growth inhibitor [17]. In addition, the dependence of iron uptake transporters on catecholamines in *E. coli* and in *S. aureus* suggests a universal siderophore function of these hormones in bacteria [12]. Growth of *Staphylococcus epidermidis* was stimulated by catecholamines [18], and norepinephrine enhanced the ability *of S. epidermidis* to inhibit *S. aureus* growth in dual-species biofilms [19]. *Micrococcus luteus* was shown to be highly sensitive to epinephrine, as reflected in a number of substantial alterations in gene expression, biofilm formation [20], biochemical composition of the biofilm matrix [21], and proteomic profiles of cells [22].

*Cutibacterium acnes* represents a unique case within microbial endocrinology. It is recognized as one of the most prevalent microorganisms on human skin [23] and particularly in acne lesions [24] and is implicated in the development of acne vulgaris. The exact mechanism of its involvement in acne development remains unclear despite a number of hypotheses (e.g., *C. acnes* phylotype-dependent dysbiosis in skin microniches or biofilm formation [24],[25]). Numerous studies have found a correlation between the stress scale and the severity of acne vulgaris in humans (e.g., [26]–[28]). *C. acnes* is known to form biofilms within skin comedones, highlighting the importance of studying its biofilms behavior [25]. However, the bacterium presents challenges due to its slow growth rate, modest biofilm production, copiotrophic nature, and sensitivity to oxygen, among other factors.

Thus, the effects of catecholamines, especially epinephrine, on *C. acnes* biofilms remain underexplored. L. Boyanova offers a comprehensive analysis of the effects of catecholamines on *C. acnes*, noting a strain-dependent hormone action, and provides an overview of catecholamine interactions with anaerobic bacteria [29]. A more recent experimental study of Borrel and co-authors focused on the direct effects of epinephrine and norepinephrine on *C. acnes* biofilms. It demonstrated that, despite the lack of visible effects on biofilm biomass growth, catecholamines (especially norepinephrine) triggered intrinsic cellular processes in *C. acnes*, resulting in shifts in the ability to stimulate sebum production in sebocytes and in cell polarity (via reduced affinity to decane in the case of *C. acnes* RT4 or to decane, hexadecane, and chloroform in the case of *C. acnes* RT6) [30]. Therefore, our study aims to characterize the intrinsic processes within *C. acnes* biofilms triggered by epinephrine at the transcriptional and translational levels. This should help to find potential targets in cells and metabolic processes affected by the hormone and suggest directions for future research on *C. acnes* as an important object of microbial endocrinology.

## 2. Materials and methods

### 2.1. Strain and cultivation

The acneic strain *Cutibacterium acnes* RT5 HL043PA2 (ATCC HM-514) was obtained from the ATCC collection (Manassas, VA, USA). The bacterium was stored long-term at −160 °C and cultured anaerobically at 33 °C on 1.5% agar reinforced clostridial medium (RCM). The cultures were refreshed every week. The composition of the liquid RCM medium was: 13 g/L yeast extract (Dia-M, Moscow, Russia), 10 g/L peptone (Dia-M, Moscow, Russia), 5 g/L glucose (Dia-M, Moscow, Russia), 5 g/L sodium chloride (Dia-M, Moscow, Russia), 3 g/L sodium acetate (Reakhim, Moscow, Russia), 1 g/L starch (Dia-M, Moscow, Russia) and 0.5 g/L L-cysteine hydrochloride (Biomerieux, Marcy-l'Étoile, France); pH 7.0. Plates containing cultures were stored in anaerobic GazPak bags (BD, Franklin Lakes, New Jersey, US) with Anaerogaz sachets (NIKI-MLT, Saint-Petersburg, Russia). To obtain the inoculum culture, the biomass of a colony was transferred into a 22 mL screw-capped glass tube filled with liquid RCM and incubated for 72 h at 33 °С. Prior to experiments, inoculates were adjusted to OD_540_ = 0.5 with sterile physiological saline (0.9% NaCl).

### 2.2. Epinephrine

Epinephrine (Merck, Darmstadt, Germany) was dissolved in sterile MilliQ water, aliquoted, and stored at −20 °C. Stock solutions were diluted in water after vigorous vortexing to obtain appropriate concentrations.

### 2.3. Biofilm growth on the PTFE cubes

Planktonic cultures and biofilms of *C. acnes* were cultured under anaerobic conditions as described by Ovcharova et al. [31]. Briefly, 21 chemically pure polytetrafluoroethylene (PTFE) cubes of size 4 × 4 × 4 mm were placed in standard 22 mL glass tubes volume with screw caps. The tubes were sealed and sterilized at 121 °C for 30 minutes. After 21 mL of RCM was added to each tube, an aliquot of epinephrine stock solution was administered in the appropriate volume and concentration. According to Boyanova, the upper limit of normal adrenaline concentration in human blood plasma is 5 nM. Therefore, the concentrations of epinephrine tested were 5 nM (normal, physiological), 50 nM (10 × physiological), 0.5 µM (100 × physiological), and 5 µM (1000 × physiological); control samples were without addition of the hormone. Each tube was then inoculated with 350 µL of *C. acnes* culture at OD_540_ = 0.5. The tubes were then sealed with screw caps and incubated at 33 °C and 150 rpm for 72 hours to obtain mature biofilms.

After incubation, the OD_540_ was measured using blank controls without bacterial inoculation, followed by biofilm crystal violet staining (CV, Sigma, St. Louis, Missouri, USA) to analyze the total amount of biofilms on the PFTE surface. To prepare the CV solution, 2.5 g of CV powder was dissolved in 10 mL of 96% ethanol, and the resulting solution was transferred to 490 mL of distilled water. Briefly, the planktonic cultures were decanted, the cubes were gently washed twice with room-temperature tap water to remove cell suspension residues, and then the biofilms were fixed with 3 mL of 96% ethanol for 20 min. After fixation, the ethanol was removed, the cubes were dried, and 3 mL of the 0.5% CV solution was added to each tube to stain the biofilms for 20 min at RT. The CV was then decanted, and the cubes were gently washed six times with tap water and replaced in new clear glass tubes. For dye extraction, 3 mL of 96% ethanol was added to each tube. The OD_590_ was measured after 90 min of extraction using blank controls. Results were calculated as relative values with the controls without epinephrine set to 100%.

### 2.4. Biofilms and planktonic cells growth for transcriptomic and proteomic analysis

For RNA isolation, biofilms were cultured on 1.5% RCM agar on glass microfiber filters of GF/F grade (GMMFs, Merck, Darmstradt, Germany). We based our investigation on the data on adrenaline concentrations in human blood plasma presented in the review by L. Boyanova [29]. To obtain biofilms, the RCM agar was melted and cooled to 55 °C to prevent hormone degradation. Then, 18 µL of an epinephrine stock solution was added to 21 mL of the melted RCM and the medium was mixed and placed in a Petri dish. After the agar solidified, a GMFF was placed in the center of a Petri dish, and 25 µL of prepared bacterial culture was inoculated onto the center of filters. The biofilms were incubated anaerobically at 33 °C for 72 h. After incubation, the biofilms on the filters were processed to extract total RNA.

For protein isolation, biofilms were cultured in 1.5% RCM agar on scaffolds of food-grade cellulose film (Ozon, Moscow, Russia). Circles of 90 mm in diameter were cut from the film, soaked in distilled water, and autoclaved at 121 °C for 30 min. Then, the cellulose circles were placed on the solidified RCM agar (one per Petri dish) with or without the addition of the hormone at the appropriate concentration (prepared as described above), and 0.5 mL of prepared inoculum was plated on the cellulose and spread with a sterile microbiological spreader. Biofilms were incubated anaerobically at 33 °C for 72 h. For each sample, bacterial biomass from 20 Petri dishes was collected and placed in a 2 mL microcentrifuge tube (Eppendorf, Hamburg, Germany). The biomass was then processed for protein isolation.

Planktonic culture was grown in 50 mL conical centrifuge tubes (Biologix, Jinan, China) filled with liquid RCM with or without the hormone. Bacteria were grown anaerobically at 33 °C for 72 h and then it was centrifuged at 4000 × g and washed twice with physiological saline. The washed pellets were collected in 2 mL microcentrifuge tubes and processed as described below.

### 2.5. RNA isolation and differential gene expression analysis

Total RNA extraction, quality control, and storage were performed as described by Ovcharova et al. [31]. Briefly, filters containing biofilms were frozen in liquid nitrogen, shredded with glass, and RNA was extracted using Qiagen RNeasy® Mini Kit (Qiagen, Hilden, Germany). To verify the correct isolation of RNA, electrophoresis was performed on 1% agarose gel with the addition of ethidium bromide embedded in a standard TAE buffer to check the quality of the RNA extraction (by visualization of two ribosomal RNA bands). Isolates were stored at −80 °C prior to sequencing.

The concentration of RNA in the samples was measured using Qubit 2.0 (Invitrogen, Waltham, MA, USA). Ribosomal RNA depletion was performed using the Illumina Ribo-Zero Plus rRNA Depletion Kit (Illumina, San Diego, CA, USA) according to the manufacturer's instructions with 120 ng of total RNA for each sample. Next, RNA libraries were prepared using the NEBNext Ultra™ II Directional RNA Library Prep Kit for Illumina (New England Biolabs® Inc., Ipswich, MA, USA) according to the manufacturer's instructions. RNA was fragmented by heating at 94 °C for 5 min. Libraries were indexed using the index primers set NEBNext Multiplex Oligos for Illumina (Dual Index Primer Set 2) from New England Biolabs® Inc., USA. Library amplification was performed in 15 PCR cycles. Reads were generated using bcl2fastq 2.20 [32] without allowing mismatches in the sequencing indices (“–barcode-mismatches = 0”).

### 2.6. Read preprocessing and quality control

The current versions of the programs used for read processing are summarized in Table 1. Read trimming was performed in Trimmomatic [33]. Reads were aligned to reference genomes (for *C. acnes* the assembly accession in the RefSeq database was GCF_000144755.1) using the Burrows-Wheeler Alignment (BWA) tool [34] with the BWA-MEM algorithm. The number of reads belonging to different genes was calculated using Salmon 1.3.0 [35] with 20 Gibbs samples and corrected for GC bias. Batch effect correction was performed using the ComBat-seq algorithm as a part of the sva library in R. Differential expression was then analyzed using DeSeq2 1.22.2 with the default parameters. Hierarchical clustering was performed using the hclust function of the R programming language with the complete linkage clustering algorithm. Principal component analysis was performed using the plotPCA function of DeSeq2.

**Table 1.** Programs and parameters for read processing.

Program	Version	Parameters applied in the study
Trimmomatic	0.39	ILLUMINACLIP: [adapters file path]:2:30:10:1:
		TRUE TRAILING:3 SLIDINGWINDOW:4:15
		AVGQUAL:20 MINLEN:30
BWA	0.7.17	
Salmon	1.3.0	–libType A –gcBias –numGibbsSamples 20
sva	3.38	
DESeq2	1.22.2	

To analyze the level of contamination in the reads, 1000 reads from each library were aligned using BLASTN 2.11.0 [36] against the NCBI nt database with a maximum e-value of 10^−5^. The taxonomy of the best BLAST hit according to the NCBI Taxonomy database was used to infer the taxonomy of the read source. The NCBI nt and NCBI Taxonomy databases were accessed on April 20, 2022. The analysis showed that the level of contamination was negligible (data not shown).

### 2.7. Quantitative PCR

To validate the results of the RNA-seq analysis for differential gene expression, qRT-PCR was additionally performed for the identified genes. Total RNA extraction was performed as previously described in at least five independent replicates. Biofilm samples for qRT-PCR were obtained independently from samples for RNA-Seq. Primers for each gene found to be differentially expressed were synthesized by Evrogen (Russia) and tested for their acceptability, including the autohybridization products. For primer testing, total DNA was extracted from planktonic cells grown for 72 h, as described above. After cell disruption in liquid nitrogen, DNA was isolated using the Magen HiPure bacterial DNA kit (Magen, Guangzhou, China). DNA was stored at −20 °C and the PCR for primer testing was performed using the Syntol (Moscow, Russia) PCR kit with standard protocol. The primers used for qRT-PCR are listed in Table 2.

**Table 2.** Primers used for qRT-PCR.

№	Gene accession	Product name	Primer sequence 5′-3′
1	HMPREF9571_RS0111905	ABC transporter substrate-binding protein	Forward TCGGAGTCACTGAACCCTCT
			Reverse GGCGTTGATGAAGTCGTTGG
2	HMPREF9571_RS03350	ABC transporter ATP-binding protein	Forward TCTAGGCCTGGTGTTGAGGT
			Reverse GACCGTCCATTCTTGGGTGT
3	HMPREF9571_RS03355	iron ABC transporter permease	Forward ACGAGCCGACAAATCACCTT
			Reverse ATGTCTGCAGTCCGTTCCAG
4	HMPREF9571_RS04615	amino acid permease	Forward AGGTCAAGGGCATCAACGAG
			Reverse AAGGAGATGGCGAACAGTGG
5	HMPREF9571_RS09715	hypothetical protein	Forward GATTTCGGGGTGTCTGTCGT
			Reverse AGAACCGGGAACTTCGTGTC
6	HMPREF9571_RS01930	CsbD family protein	Forward TTTTTCGCCGCTGTCTTGTG
			Reverse TTCCCGATTGAACTGCGACA
7	HMPREF9571_RS04360	antitoxin	Forward TCTTCGATAAGGCCAAGGACG
			Reverse CCAAGCTTGTCTTTGAGGGC
8	HMPREF9571_RS04520	DUF4193 domain-containing protein	Forward GACACGAACCGTCAACTCCT
			Reverse GCTCCGCGTAAGACTGATGA

For qRT-PCR, the synthesis of the first chain of cDNA was performed using the MMLV reverse transcriptase Kit (Evrogen, Russia) according to the manufacturer's instructions. Then, qRT-PCR was performed in PCR buffer B (Syntol, Moscow, Russia) in the presence of SYBR Green I and passive reference dye ROX for fluorescence signal normalization. 10× PCR Buffer B containing 500 mM KCl, 100 mM Tris-HCl (pH 8.8) and 0.5% (v/v) Tween 20 was diluted 10-fold and the MgCl2 was added to a final concentration of 2.5 mM. Detection was performed in duplicate for each sample. ddH_2_O (Syntol, Moscow, Russia) was used as a negative control. Amplification was performed using the PCR system CFX96 Touch TM (Bio-Rad, Hercules, CA, USA). The time and temperature profile of the reaction was as follows: polymerase activation 5 min at 95 °C; then 40 cycles of 15 s at 95 °C, 20 s at 55 °C and 40 s at 62 °C. The differential gene expression was measured in relation to the control samples (biofilms grown without CNP) as a calibrator. The *C. acnes* 16S rRNA gene was selected as a reference. The amount of a target gene was normalized to the internal control and to a calibrator identified by the comparison method Ct (ΔΔCt) as 2^-ΔΔCt^, as described by Ovcharova et al. [31].

### 2.8. Protein isolation

Each sample of biofilm or planktonic biomass was resuspended in sterile physiological saline and the suspension OD_600_ was adjusted to 1.0. Ten milliliters of the prepared cell suspension was centrifuged at 4000 × g for 30 min at room temperature. Then, the supernatant was discarded, and the pellet was resuspended in 0.6 mL of lysis buffer (Table 3). The composition of the buffer was based on the Cold Spring Harbor protocol [37], modified as described by Gannesen et al. [22]. The suspension was then transferred to 2 mL lysis tubes containing Lysing Matrix B (MP Biomedicals, Santa Ana, California, US). Biomass was disrupted for 20 s using a FastPrep disintegrator (speed regime 6) for five consecutive rounds. Tubes were then centrifuged at 13,000 × g for 1 min to precipitate the abrasive and cell debris. Protein isolation was then checked by standard Bradford assay (Sigma, Darmstadt, Germany).

**Table 3.** Composition of the lysis buffer.

Reagent	Amount	Final concentration
Tris-buffered saline (TBS; 10×, pH 7.5) – Tris Base 0.198 M, NaCl 1.51 M. pH 7.5 was adjusted by 1 M HCl	5 mL	1 ×
EDTA (0.5 M)	100 µL	0.001 M
Triton X100 10%	5 mL	1 %
Phenylmethanesulfonyl fluoride (PMSF) 10 mM in ethanol	1 mL	0.2 mM
H_2_O	38.9 mL	

### 2.9. Mass spectrometry proteomics analysis

Proteomic analysis was performed as described by Gannesen et al. [22]. Protein precipitation was performed with acetone. To a volume of cell lysate containing 20 µg of proteins, 5 volumes of cold (−20 °C) acetone (Merck, Darmstadt, Germany) were added, the mixture was vortexed and incubated overnight at −20 °C, after which the samples were centrifuged (15,000 × g at 4 °C) for 15 min, the supernatants were discarded, and the pellets were dried under vacuum at room temperature.

Reduction, alkylation, and digestion of the proteins in solution were performed as described by Kulak et al. [38], albeit with minor modifications. Sodium deoxycholate (SDC) lysis, reduction, and alkylation buffer pH 8.5 containing 100 mM TRIS, 1% (w/v) SDC, 10 mM tris(2-carboxyethyl)phosphine (TCEP), and 20 mM 2-chloroacetamide was added to a cell sample. The sample was heated at 95 °C for 10 min, and an equal volume of trypsin solution in 100 mM TRIS pH 8.5 was added at a 1:100 (w/w) ratio. After overnight digestion at 37 °C, peptides were acidified with 1% trifluoroacetic acid (TFA) for poly(styrene-divinylbenzene) reverse phase sulfonate (SDB-RPS) binding, and 20 µg was loaded on three 16-gauge StageTip plugs, an equal volume of ethyl acetate was added, and the StageTips were centrifuged at 400 × g. After washing the StageTips with 100 µL of 1% TFA/ethyl acetate mixture and 100 µL of 0.2% TFA, peptides were eluted with 50 µL of elution solution containing 50% acetonitrile, 45% water, and 5% ammonia. The collected material was vacuum dried and stored at −80 °C. Prior to analysis, peptides were dissolved in 2% acetonitrile/0.1% TFA solution and sonicated in an ultrasonic water bath for 2 min.

For the MS experiments, samples were loaded onto a handmade trap column 20 mm × 0.1 mm, packed with Inertsil ODS3 3 µm sorbent (GL Sciences, Tokyo, Japan), in the loading buffer (2% acetonitrile, 98% H_2_O, 0.1% trifluoroacetic acid) at a flow rate of 10 µL/min and separated at RT in a hand-packed [39] fused silica column 300 mm × 0.1 mm packed with Reprosil PUR C18AQ 1.9 (Dr. Maisch, Ammerbuch-Entringen, Germany) into an emitter prepared with P2000 Laser Puller (Sutter, Atlanta, GA, USA). Reverse phase chromatography was performed on an Ultimate 3000 Nano LC system (ThermoFisher, Waltham, MA, USA) coupled to a Q Exactive Plus Orbitrap mass spectrometer (ThermoFisher, USA) via a nanoelectrospray source (ThermoFisher, USA). Peptides were loaded into a loading solution (98% 0.1% (v/v) formic acid, 2% (v/v) acetonitrile) and eluted using a linear gradient: 3%–35% solution B [0.1% (v/v) formic acid, 80% (v/v) acetonitrile] for 105 min; 35%–55% B for 18 min, 55%–99% B for 0.1 min, 99% B for 10 min, 99%–2% B for 0.1 min at a flow rate of 500 nL/min. After each gradient, the column was re-equilibrated with solution A (0.1% (v/v) formic acid, 2% (v/v) acetonitrile) for 10 min. MS1 parameters were as follows: 70 K resolution, 350–2000 scan range; maximum injection time, 30 ms; AGC target, 3 × 10^6^. Ions were isolated with a 1.4 m/z window, preferred peptide match, and isotope exclusion. Dynamic exclusion was set to 30 s. MS2 fragmentation was performed in higher energy collision dissociation (HCD) mode at 17.5 K resolution with HCD collision energy of 29%, max injection time of 80 ms, and automatic gain control (AGC) target of 1 × 10^5^. Other settings were charge exclusion unassigned, 1, >7.

Raw spectra were processed using MaxQuant 1.6.6.0 (MQ) [40] and Perseus [41]. Data were searched against the *C. acnes* Uniprot Tremble database, which contains canonical and isoform proteins, using the March15, 2021 version [42].

The MaxQuant search was performed with the default parameter set, including trypsin/p-protease specificity, max 2 missed cleavages, Met oxidation, protein N-terminal acetylation, and NQ deamidation as variable modifications, and carbamidomethyl Cys as a fixed modification, max 5 modifications per peptide, 1% peptide spectrum matches (PSM,) and protein false discovery rate (FDR). The following options were enabled: second peptide, maxLFQ, and match between runs. All runs were analyzed as independent experiments and processed in Perseus.

In Perseus, the protein group results were filtered for contaminants, reverse, and “identified by site only” proteins. Only the proteins with maxLFQ values in at least 3 out of 7 LC-MS runs were used. Missing values were imputed from normal distribution with an intensity distribution sigma width of 0.3 and an intensity distribution center downshift of 1.8.

### 2.10. In silico proteome analysis

Partial visualization of protein clusters was performed using the online resource Protein–Protein Interaction Networks Functional Enrichment Analysis STRING [43]. Online protein analysis was performed using the UniProt database [42] and NCBI Protein BLAST [44].

### 2.11. Statistics

Cultivation of *C. acnes* planktonic cultures and biofilms for CV staining was performed four independent times. Non-parametric Mann-Whitney criterion was used to find significant differences, results were processed using GraphPad Prism 8.3.0, and data were presented as box and violins histogram with medians, quantiles, and all the data points. RNA preparation for RNA-Seq was performed in three biological replicates. Proteins were independently isolated in triplicate. Samples for qRT-PCR were prepared five times independently. In the RNA-seq experiments, q-values were calculated from the p-value in each experiment using a false discovery rate (FDR) correction, as proposed by Benjamini and Hochberg [45]. Significantly different proteins were detected using Prism GraphPad 8.3.0. Multiple t-test with the additional parameter “SD assuming (more power)” was used. FDR correction was performed using the “two-stage step-up integrated method of Benjamini, Krieger and Yekutieli” parameter [46].

## 3. Results

### 3.1. Biofilm growth on the PTFE cubes

**Figure 1.**
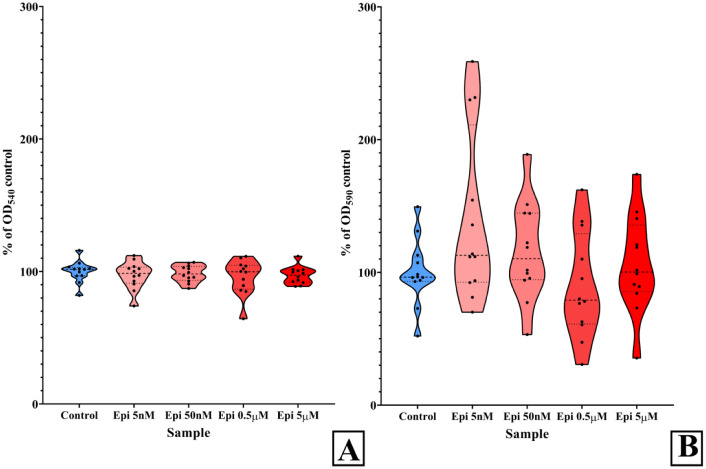
Effects of epinephrine on planktonic cultures (A) and biofilms (B) of *C. acnes* HM514 after 72 h of incubation. Values from four independent replicates, each containing three technical replicates, are plotted points on the graph.

First, we analyzed the planktonic growth of *C. acnes* after 72 h of incubation. In planktonic cultures, the variation in values was significantly lower than in biofilms (Figure 1), which is normal for CV staining. Nevertheless, epinephrine had no significant effect in either planktonic cultures or biofilms. We could say that the hormone slightly stimulated biofilm growth only at 5 nM (the minimum value was 70%, the maximum value was 258.9%, the median value was 111% of the control), but the p-value = 0.0563 did not allow to consider this difference as significant.

Since Borrel and co-authors obtained similar results [22], we decided to go deeper and perform the molecular study of intrinsic epinephrine effects on *C. acnes* using transcriptomic and proteomic analysis.

### 3.2. Study of differential gene expression

In total, 2498 genes were identified in *C. acnes* strain HM514. After CV staining of biofilms, in transcriptomic and proteomic experiments we decided to test epinephrine at concentrations of 5 nM (as normal in human blood plasma) and 5 µM to simulate stress conditions. First, we analyzed how epinephrine affects gene expression in *C. acnes* biofilms at concentrations of 5 nM and 5 µM compared with samples without the hormone and assessed the differences between the two groups of hormone-treated samples. In biofilms at the lower concentration (5 nM), no differentially expressed genes were detected compared with controls without epinephrine. At the higher concentration, epinephrine downregulated five genes compared with controls without hormone addition (Table 4), four of which were genes of transporter proteins. Comparison between the epinephrine-treated samples revealed downregulation of four genes at the higher concentration of 5 µM compared to samples at the concentration of 5 nM (Table 4), and the putative permease HMPREF9571_RS04615 (ALT35263.1) was downregulated with a similar fold change as in the previous sample pair. Therefore, at least this gene can be considered as a putative target for epinephrine-mediated regulation. These data suggest that the effects of epinephrine are dependent on the hormone concentration.

In addition, protein analysis was performed to determine which genes were downregulated in 5 µM samples compared with controls without the hormone. STRING analysis revealed the cluster of FecCD family proteins (ALT35045.1, ALT35043.1, and ALT35044.1), which are responsible for Fe(III) uptake [47]. Thus, the downregulation of Fe(III) uptake protein genes may potentially be a *C. acnes* cell response to an additional siderophore in the medium. However, as we studied only the endpoint cell status, a deeper investigation of proteomic and transcriptomic profiles of *C. acnes* in dynamics should be performed in the future.

### 3.3. Quantitative RT-PCR

Quantitative RT-PCR was performed to validate the RNA-Seq results. Five independent RNA samples were obtained for each condition, and qRT-PCR was performed for each differentially expressed gene for pairwise comparison. Comparison of 5 µM epinephrine samples with controls (Table 4) showed that RNA-Seq data were in agreement with qRT-PCR in four replicates, while the last replicate was not in agreement with RNA-Seq results. However, we can assume that, in general, the effects of 5 µM epinephrine versus control were highly reproducible.

To validate the differential gene expression in 5 µM epinephrine samples vs. 5 nM samples, we performed the qRT-PCR of corresponding genes and the qRT-PCR of genes downregulated in 5 µM epinephrine samples versus 5 nM epinephrine samples. We measured the gene expression in comparison with control samples and calculated the log_2_ of the ratio between gene expression in both sample groups (Table 5). We found that, in general, qRT-PCR validated the results of RNA-Seq between two epinephrine sample groups. However, the differential expression of genes not detected by RNA-Seq in 5 nM epinephrine samples varied significantly. This potentially means that 5 nM may be close to the threshold for affecting the gene expression, and a more granular approach may reveal the dose-response relationship of epinephrine.

**Table 4.** Differential gene expression in *C. acnes* in presence of epinephrine.

Sample comparison	Locus tag (in brackets – protein accession # on STRING)	Product name	Log2 (expression level ratio)	Standard error of log2 (expression LEVEL ratio)	p–value	q–value	Conclusion
*С. acnes* epinephrine 5 µM compared to *С. acnes* control	HMPREF9571_RS0111905 (ALT35045.1)	ABC transporter substrate-binding protein	−3.36	0.57	3.5 × 10^−5^	0.0244	lower
	HMPREF9571_RS03350 (ALT35043.1)	ABC transporter ATP-binding protein	−2.85	0.45	3.9 × 10^−5^	0.0244	lower
	HMPREF9571_RS03355 (ALT35044.1)	Ferrichrome iron ABC transporter permease	−2.66	0.42	9.29 × 10^−5^	0.0464	lower
	HMPREF9571_RS04615 (ALT35263.1)	Putative aromatic amino acid permease	−2.99	0.45	1.21 × 10^−5^	0.0244	lower
	HMPREF9571_RS09715 (ALT36370.1)	Hypothetical protein	−2.64	0.40	3.71 × 10^−5^	0.0244	lower
*C. acnes* epinephrine µM compared to *С. acnes* epinephrine 5 nM	HMPREF9571_RS01930 (ALT35563.1)	Putative stress response protein (CsbD family)	−3.01	0.40	5.77 × 10^−7^	0.0014	lower
	HMPREF9571_RS04360 (ALT35217.1)	Putative antitoxin	−2.94	0.46	2.57 × 10^−5^	0.0237	lower
	HMPREF9571_RS04520 (ALT35247.1)	DUF4193 domain-containing protein– dUTPase	−2.73	0.44	7.15 × 10^−5^	0.0446	lower
	HMPREF9571_RS04615 (ALT35263.1)	Putative aromatic amino acid permease	−2.90	0.45	2.85 × 10^−5^	0.0237	lower

**Table 5.**
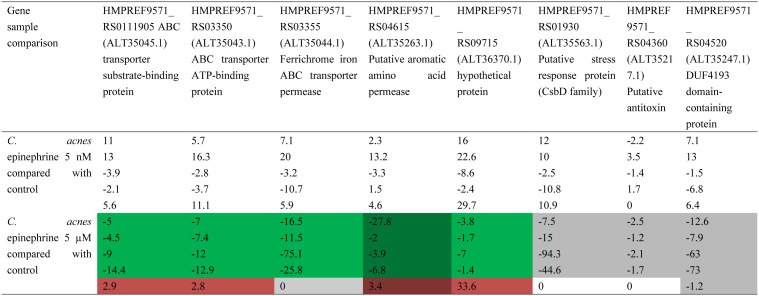
Quantitative RT-PCR results for genes detected in RNA-Seq. Green stands for correspondence between qRT-PCR and RNA-Seq data in comparison of 5 µM epinephrine and control samples where it is applicable. Gray stands for no differential expression, dark pink means discordance between qRT-PCR and RNA-Seq data in comparison of 5 µM epinephrine and control samples where it is applicable. Shaded cells stand for correspondence between qRT-PCR and RNA-Seq data in comparison of 5 µM and 5 nM epinephrine samples.

These results suggest that epinephrine affects the gene expression in *C. acnes* biofilms. However, it is unclear why the downregulated genes are different in the two samples groups. Potentially, a multi-target manner of epinephrine action could explain this phenomenon. However, just as in *M. luteus* [20], the mechanistic targets of epinephrine remain elusive.

### 3.4. Proteomics changes in *C. acnes* planktonic cultures caused by epinephrine in comparison with control samples

A total of 1306 proteins were identified in both planktonic cultures and biofilms of *C. acnes*. First, we tested how the hormone affected the planktonic cultures of *C. acnes* at a lower concentration impact. Only two proteins were affected by epinephrine, both of which are involved in vitamin synthesis (Table 6). Thiamine monophosphate kinase was downregulated, which could lead to an alteration in thiamine processing, while biotin synthase ALT35548.1 was upregulated. The latter one was the same enzyme that was upregulated in control biofilms compared with control planktonic cultures (see below). We propose that this enzyme is a potential target for the hormone, and its upregulation by both epinephrine and biofilm formation is further evidence that epinephrine mimics the changes that occur upon in biofilms.

At a higher concentration, 5 µM epinephrine altered four proteins in planktonic cultures (Table 7). No biotin synthase was detected, but all three proteins downregulated by the hormone were downregulated in a similar manner as in control biofilms versus planktonic cultures (see below). Hence, at higher concentrations, epinephrine also *erases* differences between planktonic cultures and biofilms, probably making planktonic cells more *biofilm-like*.

Finally, we compared the planktonic samples treated with 5 nM epinephrine and with 5 µM epinephrine. This comparison revealed more differences than between epinephrine and control samples (Table 8). It is noteworthy that four proteins (ATP-dependent DNA helicase of the RecQ family, biotin synthase AOG29170.1, bifunctional glutamine synthetase adenylyltransferase, and biotin synthase ALT35548.1) were the same as in the list obtained from the untreated control cultures. However, while three of the aforementioned proteins were downregulated by epinephrine at the higher concentration as well as by biofilm formation, ALT35548.1 was downregulated instead of upregulated in control biofilms (see below). Thus, these facts demonstrate the multi-target nature of epinephrine action and provide additional evidence for the hypothesis of equalization between planktonic cultures and biofilms caused by epinephrine at a higher concentration in the medium.

### 3.5. Proteomics changes in *C. acnes* biofilms caused by epinephrine in comparison with control samples

In biofilms, the hormone at a lower concentration altered the levels of only three proteins (Table 9). The amino acid permease ALT35254.1 was downregulated by epinephrine, as it was downregulated in control biofilm samples compared with planktonic cultures (see below). Putative exodeoxyribonuclease V, gamma subunit was upregulated in biofilms in the presence of 5 nM epinephrine, whereas it was downregulated in planktonic cultures by 5 µM epinephrine (Table 9). Finally, the D-alanine-D-alanine ligase ALW21_11825 was upregulated by 5 nM epinephrine in biofilms. This protein was downregulated in control biofilms compared with control planktonic cultures (see below). No genes were found to be differentially expressed in the presence of 5 nM epinephrine.

**Table 6.**
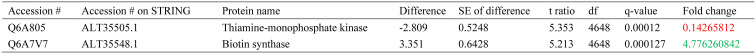
Proteomic changes in *C. acnes* planktonic cultures in the presence of 5 nM epinephrine in comparison with control. Red color indicates downregulated proteins. Green color indicates upregulated proteins.

**Table 7.**
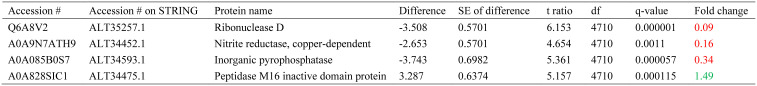
Proteomic changes in *C. acnes* planktonic cultures in the presence of 5 µM epinephrine in comparison with control. Red color indicates downregulated proteins. Green color indicates upregulation.

**Table 8.**
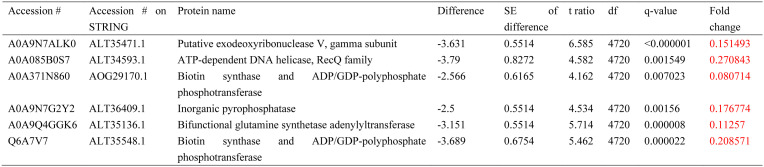
Proteomic changes in *C. acnes* planktonic cultures in the presence of 5 µM epinephrine in comparison with 5 nM epinephrine. Red color indicates downregulated proteins.

At the higher (5 µM) concentration, the number of proteins affected by the hormone was higher (Table 10) in biofilms compared with control samples, but no matches between differentially expressed genes and proteins were detected. Among the downregulated proteins, two clusters were detected using STRING. The first consists of two PTS sugar transport proteins (ALT34950.1, ALT34951.1, N-acetylglucosamine transport), suggesting the downregulation of N-acetylglucosamine translocation. The second cluster includes proteins ALW21_09320 (ribosomal) and the elongation factor EfP (ALW21_05985), suggesting some alteration in translation processes. Among the upregulated proteins, the cluster of two enzymes (RecBCD enzyme subunit RecB ALT35472.1 and exodeoxyribonuclease V gamma subunit ALT35471.1) are involved in the repair of double-strand breaks by homologous recombination (according to KEGG). Therefore, it may indicate increased DNA stability in *C. acnes*. The following proteins are of special interest. First, the amino acid permease ALT35254.1 was downregulated by 5 µM epinephrine, as was shown for 5 nM epinephrine in the medium. Second, the exodeoxyribonuclease V, gamma subunit was upregulated, as it was shown for the lower hormone concentration. Peptidase M16 inactive domain protein ALT34475.1 was the same as it was in planktonic cultures in the presence of 5 µM epinephrine. Finally, the RecBCD enzyme subunit RecB was downregulated in control biofilms compared to planktonic cultures (see below), and 5 µM of the hormone upregulated it in the biofilms. Several other proteins were altered in their concentrations in the presence of the hormone (Table 10).

**Table 9.**
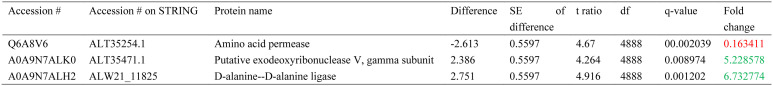
Proteomic changes in *C. acnes* biofilms in the presence of 5 nM epinephrine in comparison with control. Red color means downregulated proteins. Green color means upregulated proteins.

**Table 10.**
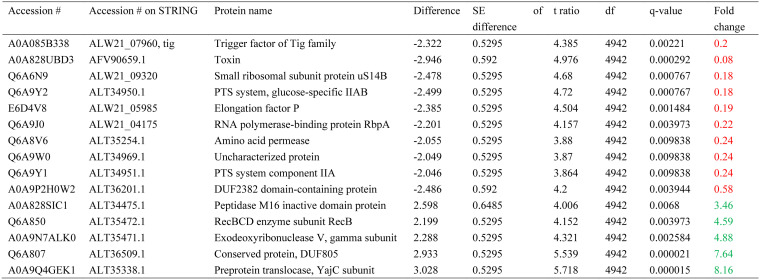
Proteomic changes in *C. acnes* biofilms in the presence of 5 µM epinephrine in comparison with control biofilm samples. Red color means downregulated proteins. Green color means upregulated proteins.

Comparing the biofilms grown in the presence of 5 nM and 5 µM of epinephrine, no proteomic difference was detected, although the four genes were downregulated at the higher epinephrine concentration.

### 3.6. Proteomic alterations between *C. acnes* planktonic cultures and biofilms: how epinephrine impacts the difference between planktonic cells and biofilms

**Figure 2.**
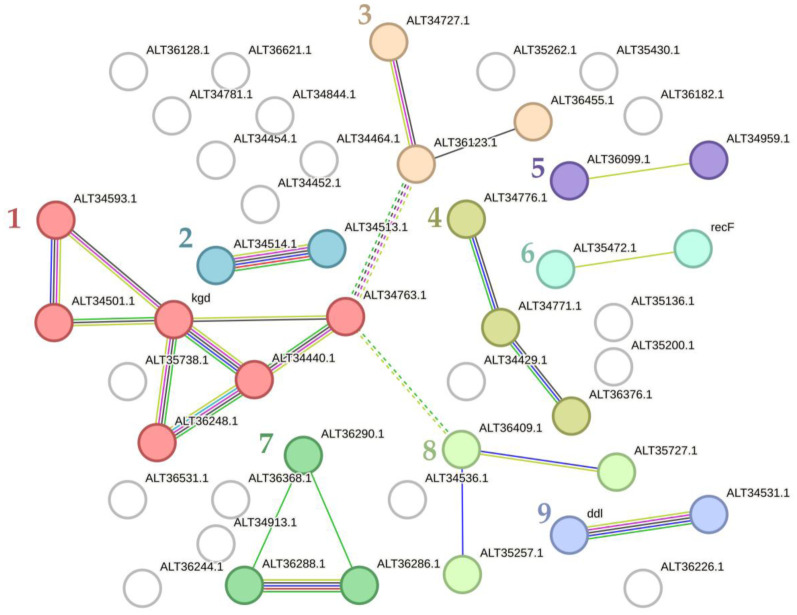
Downregulated proteins in *C. acnes* biofilms in comparison with planktonic cultures (in control samples). 1 (Red)–TCA and propanoate metabolism, 2 (turquoise)–Fe-S proteins, transmembrane transport of hydroxyl organic compounds; 3 (beige)–oxidoreductases, 1 trehalose phosphatase; 4 (khaki)–inositol and sugars metabolism; 5 (purple)–fimbrial protein (CAMP factor Cfa) and triacylglycerol lipase (cell envelope?), host interaction; 6 (aquamarine)–DNA processing and repair, 7 (light green)–glycerol dehydrogenase and ribulokinase activity, pentose phosphate pathway, and amino acid synthesis; 8 (mint)–host immune system evasion, amino group processing, and regulation; 9 (pale cornflower blue)–cell wall synthesis (including FtsI). White color represents non-clustered proteins.

In control samples, a total of 48 proteins were downregulated and three were upregulated (Table S1). We visualized and clustered the downregulated proteins MCL clustering with inflation parameter 3) using the STRING online software (version 12.0 of July 26, 2023) (Figure 2). Similar to *M. luteus* [22] in *C. acnes* biofilms, proteins of the tricarboxylic acid (TCA) cycle and propanoate metabolism were partially downregulated with alpha-ketoglutarate decarboxylase Kdg being the “core” protein of the TCA cluster (cluster 1 in Figure 2). Next, it was detected the inositol metabolism cluster (cluster 4 in Figure 2) and the cluster of proteins hypothetically responsible for host immune system evasion, exopeptidase activity, and dinitrosyl-Fe binding (cluster 8 in Figure 2). In addition to the eight cluster, there was a decrease in CAMP-factor and lipase, which are also responsible for host–bacterial interactions and pathogenesis [48]. Therefore, we can assume that at least some virulence factors are reduced in *C. acnes* biofilms compared to planktonic cells. Some Fe-S-containing proteins, including Fe-S oxidoreductase (ALT34513.1), proteins of the pentose phosphate pathway, and proteins involved in amino acid synthesis and sugar metabolism were downregulated. Several transport proteins and proteins involved in DNA repair (cluster 6 in Figure 2) were found at lower levels in biofilms compared with planktonic cells. Interestingly, some proteins involved in cell wall synthesis (D-alanine-D-alanine ligase Ddl, cluster 9 in Figure 2) and cell division (FtsI) were downregulated in biofilms as well as in *M. luteus* [22], suggesting the universality of this phenomenon. Some other proteins with different functions, including zinc-binding dehydrogenase ALT36226.1 and CAMP factor Cfa ALT34959.1, were also downregulated.

Three proteins were upregulated in control biofilms compared to planktonic cultures: biotin synthase, conserved phage-associated protein, and amino acid permease. Interestingly, ALT35254.1, which is reported to be an amino acid permease and DNA binding protein, was upregulated at the same time that some proteins involved in amino acid synthesis were downregulated. This may reflect a cellular response in biofilms to maintain the amino acid balance. Furthermore, upregulation of biotin synthase (and ADP/GDP-polyphosphate phosphotransferase) ALT35548.1 may result in increased biotin synthesis and hence in increased catabolism of fatty acids [49]. *C. acnes* is known to prefer sebaceous areas of the skin and to form biofilms in sebaceous glands and hair follicles [25]. Therefore, this may serve as indirect evidence for an adaptation to such a lifestyle.

**Figure 3.**
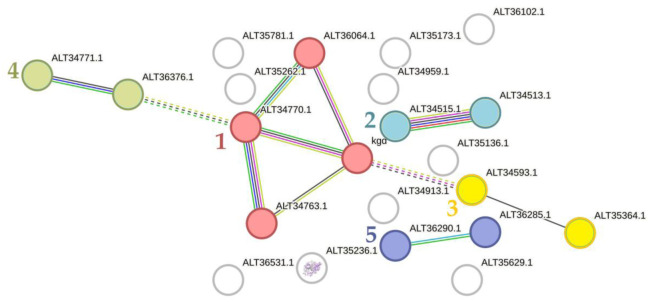
Downregulated proteins in *C. acnes* biofilms in comparison with planktonic cultures (in 5 nM epinephrine samples). 1 (red)–TCA; 2 (turquoise)–Fe-S protein and lactate utilization protein C, transmembrane transport of hydroxyl organic compounds; 3 (yellow)–ribosomal protein and inorganic pyrophosphatase; 4 (khaki)–inositol and sugars metabolism; 5 (purple)–ribose-5-phosphate processing. White color indicates non-clustered proteins.

We then compared planktonic cultures and biofilms grown in the presence of 5 nM epinephrine in the medium. Interestingly, only 24 proteins were downregulated, and three were upregulated in biofilms. Furthermore, these proteins did not correspond to those differentially present in control cultures (Table S2). A cluster of TCA and propanoate metabolism proteins (cluster 1 in the Figure 3, only alpha-ketoglutarate decarboxylase Kgd was the same as in control samples), some sugar processing proteins, and another CAMP factor 3 ALT36102.1 were among the downregulated proteins (Figure 3). Interestingly, lipase ALT36099.1 was not downregulated in 5 nM epinephrine-treated biofilms in contrast to control samples, indicating that this enzyme is present in higher concentration in epinephrine-treated biofilms. Therefore, epinephrine may modulate the virulence of *C. acnes* at the lower concentration of 5 nM. Also, the cluster of proteins of inositol metabolism (cluster 4 in Figure 3), similar to the previous figure, was downregulated. Thus, the alterations in inositol metabolism were partially maintained in epinephrine-treated biofilms. Of note, no cell division proteins were downregulated in biofilms in the presence of epinephrine, which is consistent with the hypothesis of “acceleration” of some processes in biofilms in the presence of epinephrine, as suggested for *M. luteus* by Gannesen et al. [22]. Among the upregulated proteins, the ATP-dependent DNA helicase of the RecQ family ALT36130.1 is of interest because its upregulation may lead to improved DNA repair, which is necessary for biofilm maintenance.

The proteomic analyses revealed that epinephrine at lower concentration partially eliminated the differences between *C. acnes* planktonic cultures and biofilms. When we compared biofilms and planktonic cultures in the presence of the higher concentration of epinephrine (5 µM), there were no significant differences in the proteomic profiles. 5 µM of epinephrine made the biofilms and planktonic cultures completely identical. This phenomenon is of particular interest, because in the case of *M. luteus*, hundreds of proteins were altered in biofilms compared with planktonic cultures [22].

**Figure 4.**
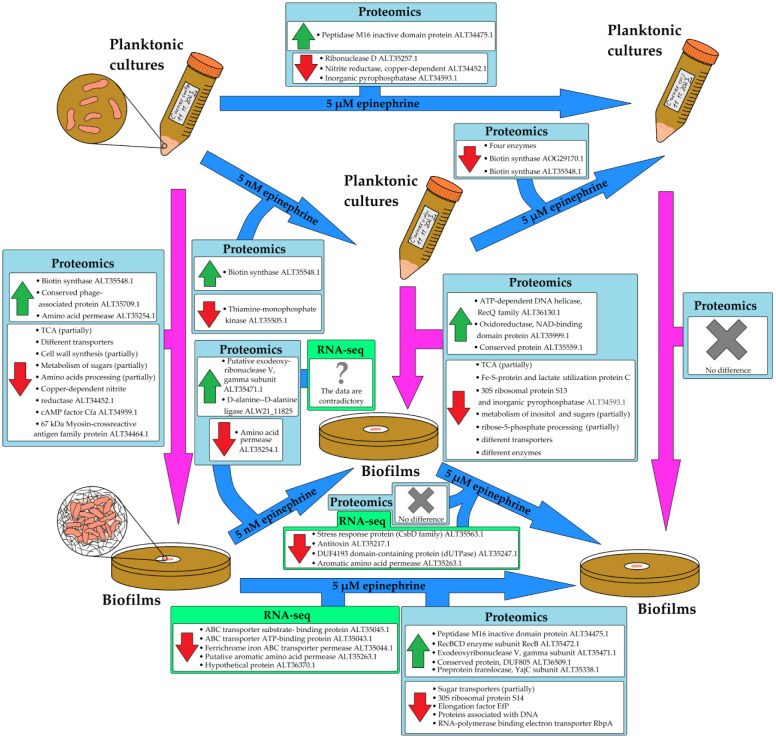
General scheme of epinephrine action on planktonic cultures and biofilms. Blue arrows show differences between control, 5 nM epinephrine, and 5 µM epinephrine samples. Magenta arrows indicate differences between planktonic cultures and biofilms.

Epinephrine has a complex effect on *C. acnes*. Analyzing the overall proteomic changes (Figure 4) between planktonic cultures and biofilms, and how epinephrine affects the bacterium in both life forms, we can summarize that the effect of epinephrine is dose-dependent and is strongest at the higher concentration in both planktonic cultures and in biofilms. At the same time, fewer proteomic changes were detected between samples grown at different epinephrine concentrations. There was no agreement between gene expression profiles and proteomic profiles in biofilms. This may be due to differences in the dynamics of cell response to the hormone at the translational and transcriptional levels and/or to multi-target effects of the hormone. Epinephrine treatment makes planktonic cultures *more similar* to biofilms, at least in their proteomic profiles. At the higher concentration, the difference in proteomic profiles between planktonic cultures and biofilms disappeared.

Regarding the more specific mechanisms and cellular targets for epinephrine, we can suggest that Kgd, biotin synthase ALT35548.1, amino acid permease ALT35254.1, gamma subunit of exodeoxyribonuclease V ALT35471.1, and RecBCD enzyme subunit RecB ALT35472.1 are among the key targets for epinephrine action on the translational level in the conditions used in our experiments (72 h of incubation). It is also important to note the downregulation of gene expression for three proteins of Fe(III) uptake. This is an interesting phenomenon due to the involvement of epinephrine as a xenosiderophore and thus due to a possible response of *C. acnes* cell to the presence of an additional siderophore in the medium. At the transcriptional level, the changes in gene expression may be a result of complex intracellular processes in biofilms that led to the downregulation of transporter protein genes. It is also important to note that the proteomic and transcriptomic profiles of cells in biofilms suggest the general decrease of metabolic rate in biofilms and especially in planktonic cultures in the presence of epinephrine.

## 4. Discussion

The primary goal of this study was to identify catecholamine targets in *C. acnes* for future in-depth investigation. The relationship between acne vulgaris and fluctuations in sex hormone level is well established [50], and some data are now available on how the stress hormones affect *C. acnes* [29],[30]. We focused our efforts primarily on *C. acnes* biofilms because this lifestyle appears to be predominant for bacteria especially in the skin microenvironment [51], and *C. acnes* biofilms remain a relatively understudied object. Despite the lack of significant differences between hormone-treated and untreated *C. acnes* planktonic cultures, shown by the CV staining method, intrinsic processes induced by epinephrine were revealed using omics techniques.

The lack of concordance between transcriptomic and proteomic profiles of *C. acnes* biofilms was not surprising. Transcription and translation are known to be regulated by different systems, and the discrepancy between the mRNA levels and proteins is a common phenomenon [52]. For a compound like epinephrine, which acts on a variety of cellular targets, this discrepancy was to be expected. Furthermore, due to the lower growth rate of *C. acnes*, 72 h is a sufficient time interval for biofilms formation and biomass accumulation. This ensures a sufficient yield of protein and RNA extraction for the analysis. However, by waiting for biomass accumulation, we lose information on the epinephrine-mediated changes in gene expression and proteomic profiles on a shorter time scale. Therefore, it is preferable to perform the analysis in a dynamic manner, as was done, for example, in a recent study by Bakker et al. on *Folsomia candida* [53]. However, we were challenged by the slow growth and lower biomass yields, which may require larger volumes of medium, a different type of biofilm carrier, and other modifications to the experimental procedure. It is a matter of future research to determine the exact changes in *C. acnes* cells in the presence of epinephrine. What can we conclude so far about the mechanisms of epinephrine action on *C. acnes* and its biofilms? First of all, we must emphasize that the effect of epinephrine appears already at the lower concentration, close to physiological in human blood plasma [29]: 5 nM. Despite the lack of significant differences in gene expression, the “residual” proteomic differences suggest the potential transcriptomic changes at the initial growth stages. The differences between the effects of epinephrine at different concentrations suggest the existence of multiple targets responding to the hormone in the cells. The most interesting question is why no differences between planktonic cultures and biofilms were detected at the higher concentration of the hormone. Does this mean that biofilms became “*more planktonic*” or (more likely), that planktonic cultures became “*more biofilm*-like”? We suspect that this is a mutual phenotypic approximation, but it should be demonstrated in further experiments with *C. acnes* biofilms.

Regarding the molecular mechanisms, we have shown that the response of mature cultures and biofilms of *C. acnes* to epinephrine is rather weak and “steady” compared to *M. luteus* [20],[22] and, for example, to the *A. hydrophila* reaction to norepinephrine [7]. In *A. hydrophila*, hundreds of genes were altered in expression, and dozens of proteins were regulated by the hormone. In *M. luteus*, seven genes and dozens or hundreds (depending on the different factors) of proteins were affected by epinephrine. In other microorganisms, such as *E. coli*, more than 500 genes were regulated by norepinephrine [54]. Thus, epinephrine has a less global but still significant effect in *C. acnes*. Despite the scattered and non-obvious molecular pathways, something can be predicted. First, considering the recent data in *M. luteus*, the TCA cycle seems to be regulated by epinephrine in actinobacteria. *C. acnes* is able to use the TCA cycle, and some proteins in *C. acnes* are moonlighting in the TCA cycle in parallel with their main function [55]. Therefore, epinephrine effects on such an important source of electron carrier molecules as the TCA cycle may potentially lead to global changes in cellular metabolism.

Next, epinephrine may affect transport processes in *C. acnes*, resulting in both differential gene expression and proteomic profiles. This is very similar to the findings made in *A. hydrophila* response to norepinephrine [7]. Thus, one type of the processes altered by catecholamines in bacteria are transport systems. Differences between genes and proteins downregulated by the hormone reflect the complexity of the catecholamine-mediated metabolic regulation in *C. acnes* and warrant investigation of time-dependence of catecholamine effects. Cell division process and cell wall synthesis may also be targets of epinephrine in *C. acnes* and in actinobacteria [22].

Third, the siderophore function of catecholamines is well known [22],[56], and the downregulation of genes of Fe(III) uptake proteins allows suggesting some regulatory processes in *C. acnes* at least after 72 h of incubation caused by xenosiderophore epinephrine. Because of the mismatch between proteomic and transcriptomic profiles after 72 h, in the future the analysis of the status of *C. acnes* cells in dynamics with at least several time points should be provided. It would elucidate whether there are immediate regulatory effects of epinephrine (initial stages of growth) or long-term late effects (more than 72 h), and how exactly the proteomic and transcriptomic profiles correlate. Nevertheless, changes in the uptake of Fe, a critical trace constituent, can lead to various consequences in cells. Therefore, the indirect epinephrine effects may also be based on this process.

Fourth, the decrease of some virulence factors such as CAMP-factor in biofilms compared with planktonic cultures (in controls and in the presence of 5 nM epinephrine) allows suggesting that epinephrine potentially modulates the virulence of *C. acnes* biofilms even at the lower concentration. No difference between planktonic and biofilm proteomes at the higher epinephrine concentration suggests an increase in *C. acnes* virulence in the presence of the hormone, which should be validated in the future. This is also indirectly consistent with the increased amount of some recombination proteins in biofilms in the presence of 5 µM epinephrine. If these proteins are involved in DNA repair, cells in biofilms may be more resistant to DNA damage, but this should also be validated in the future.

Finally, in *C. acnes*, biotin synthase and biotin-associated cellular processes are also potentially regulated by epinephrine. In a recent study, no significant effect of epinephrine on *C. acnes* RT4 and RT6 biofilm biomass was demonstrated, but some deeper processes in *C. acnes* cells led to an increase in its ability to stimulate lipid production in sebocytes [30]. This indirectly correlates with our findings of biotin synthase upregulation by epinephrine. Biotin is involved in fatty acid metabolism, hence the *C. acnes* properties described by Borrel et al. [30], and our findings may be related. In summary, through these findings we see clear similarities to the recently described epinephrine-induced changes in *M. luteus* [22]. Therefore, we can tentatively suggest that at least the TCA cycle and cell division processes are universal targets for epinephrine in actinobacteria. What are the prospects? It is necessary to delve deeper into the behavior of *C. acnes* biofilms in the presence of epinephrine and detect all the changes caused by the hormone, using recombinant strains for validation. Next, it is important to study the transcriptomic and proteomic alterations in *C. acnes* in time-resolved experiments to obtain a detailed picture of cellular dynamics in the presence of epinephrine in both planktonic and biofilm conditions. Finally, it is crucial to identify and experimentally validate the molecular targets of epinephrine in *C. acnes* cells. We can use molecular docking to predict the potential epinephrine receptor protein [8], but this can only be done if all the potential targets are identified.

Microbial endocrinology is a novel and complex discipline, and most phenomena are yet to be discovered. Our present findings in *C. acnes* response to epinephrine should provide hypotheses for future testing.

## 5. Conclusions

We have demonstrated the intrinsic effect of epinephrine on *C. acnes* biofilms and planktonic cultures. Despite the absence of visible changes in CV-stained biofilms, several genes were differentially expressed and many proteins were affected in the presence of the hormone, which may be a reason for the changes in *C. acnes* behavior reported previously. The effect of the hormone depends on the concentration, so all these data suggest the multi-target effect of epinephrine on cutibacteria. Furthermore, epinephrine "erases" the difference between the proteomic profiles of planktonic cultures and biofilms of C. acnes, and the potential targets of the hormone are the tricarboxylic acid cycle, biotin synthesis, cell division, and transport of various compounds. These processes are potentially the universal targets of epinephrine in actinobacteria.


