# Supplementary material for: Proteomic and transcriptomic analyses of Cutibacterium acnes biofilms and planktonic cultures in presence of epinephrine
Source: AIMS Microbiol. 2024 May 27;10(2):363–90. doi: 10.3934/microbiol.2024019 (PMC11194618; doi:10.3934/microbiol.2024019)
Supplement: Supplementary file 1 [file microbiol-10-02-019-s001.pdf]

AIMS Microbiology, 10(2): 363–390.
DOI: 10.3934/microbiol.2024019
Received: 04 March 2024
Revised: 15 May 2024
Accepted: 21 May 2024
Published: 27 May 2024

http://www.aimspress.com/journal/microbiology

Research article

Proteomic and transcriptomic analyses of *Cutibacterium acnes* biofilms and planktonic cultures in presence of epinephrine

Gannesen AV[1,*], Schelkunov MI[2,3], Ziganshin RH[4], Ovcharova MA[1], Sukhacheva MV[1], Makarova NE[2], Mart'yanov SV[1], Loginova NA[1], Mosolova AM[1,5] Diuvenji EV[1], Nevolina ED[1] and Plakunov VK[1]

[1] Federal Research Centre "Fundamentals of Biotechnology" of Russian Academy of Sciences, Moscow 119071, Russia

[2] Skolkovo Institute of Science and Technology, Moscow 121205, Russia

[3] Institute for Information Transmission Problems of Russian Academy of Sciences, Moscow 127051, Russia

[4] Institute of Bioorganic Chemistry, Russian Academy of Sciences, Moscow 117997, Russia

[5] Russian Biotechnological University, Moscow 125080, Russia

* **Correspondence:** andrei.gannesen@gmail.com.

Supplementary

Table S1. Proteomic changes *in C. acnes* in control biofilms in comparison with control planktonic cultures. Red color indicates downregulated proteins. Green color indicates upregulated proteins.

Accession #	Accession # on STRING	Protein name	Difference	SE of difference	t ratio	df	q value	Fold change
A0A085B0S7	ALT34593.1	Inorganic pyrophosphatase	−2.895	0.6159	4.701	4834	0.000147	0.12
A0A9Q4C1B0	ALT36376.1	Oxidoreductase, NAD-binding domain protein	−6.287	0.6159	10.21	4834	<0.000001	0.02
Q6A5D5	ALT36290.1	Ribose 5-phosphate isomerase	−4.736	0.5509	8.597	4834	<0.000001	0.04
A0A9N7ATH9	ALT34452.1	Nitrite reductase, copper-dependent	−4.513	0.5509	8.192	4834	<0.000001	0.04
Q6AAK0	ALT34771.1	Sugar phosphate isomerase/epimerase	−4.408	0.5509	8.003	4834	<0.000001	0.05
Q6A8V2	ALT35257.1	Ribonuclease D	−3.898	0.5509	7.076	4834	<0.000001	0.07
E6D3P8	ALT34776.1	Oxidoreductase, NAD-binding domain protein	−4.234	0.6159	6.875	4834	<0.000001	0.07
A0A828SGK0	ALT35727.1	Aminopeptidase N	−3.574	0.5509	6.489	4834	<0.000001	0.08
A0A9Q4GDG5	ALT34531.1	Penicillin-binding protein, transpeptidase domain protein	−2.587	0.6159	4.201	4834	0.001144	0.09
A0A371N860	AOG29170.1	ADP/GDP-polyphosphate phosphotransferase	−3.582	0.6159	5.817	4834	<0.000001	0.09
A0A9Q4BZN0	ALT34464.1	67 kDa Myosin-crossreactive antigen family protein	−3.82	0.6159	6.203	4834	<0.000001	0.10
A0A9N7AVS8	ALW21_06360	Oxoglutarate dehydrogenase (Succinyl-transferring), E1 component	−3.26	0.5509	5.919	4834	<0.000001	0.10
A0A9Q4BZZ3	ALT36368.1	Hyaluronate lyase	−2.364	0.6159	3.839	4834	0.00418	0.12
A0A9Q4BXX9	ALT36531.1	Bacterial extracellular solute-binding protein	−3.065	0.5509	5.563	4834	0.000003	0.12
Q6ABE1	ALT34514.1	Iron-sulfur protein	−3.008	0.5509	5.461	4834	0.000005	0.12
A0A9N7G0V0	ALT34913.1	Amidinotransferase	−2.944	0.5509	5.344	4834	0.000008	0.13
A0A9Q4C050	ALT35200.1	Carbamoyl-phosphate synthase large chain	−2.843	0.5509	5.16	4834	0.00002	0.14
A0A9N7G2Y2	ALT36409.1	Bifunctional glutamine synthetase adenylyltransferase/adenylyl-removing enzyme	−2.817	0.5509	5.113	4834	0.000025	0.14
Q6AAJ0	ALT34781.1	Conserved protein, putative helicase of DeaD/DeaH box family	−2.779	0.5509	5.044	4834	0.000033	0.15
Q6ABE2	ALT34513.1	Conserved protein	−2.766	0.5509	5.021	4834	0.000036	0.15

Continued on next page

Accession #	Accession # on STRING	Protein name	Difference	SE of difference	t ratio	df	q value	Fold change
A0A8B2VQT6	ALT34454.1	N-acetyltransferase	−2.68	0.5509	4.864	4834	0.000075	0.16
E6D4M0	ALT36128.1	DEAD/DEAH box helicase	−2.643	0.5509	4.798	4834	0.0001	0.16
A0A828SLV1	ALT36244.1	Alanine dehydrogenase	−2.614	0.5509	4.744	4834	0.000124	0.16
A0A9N7B064	AOG29020.1	Pyridine nucleotide-disulfide oxidoreductase	−2.373	0.6159	3.853	4834	0.004135	0.17
Q6ABF6	ALT34501.1	NADH dehydrogenase	−2.543	0.5509	4.616	4834	0.000212	0.17
Q6AAL0	ALT34763.1	Transaldolase	−2.432	0.5509	4.415	4834	0.000523	0.19
A0A9N7AN67	ALT35430.1	DoxX family protein	−2.371	0.5509	4.304	4834	0.000835	0.19
A0A9Q4GGK6	ALT35136.1	ATP-dependent DNA helicase, RecQ family	−2.333	0.5509	4.236	4834	0.001081	0.20
A0A9N7FZU1	ALT34959.1	cAMP factor (Cfa)	−2.326	0.5509	4.223	4834	0.001081	0.20
A0A9N7AS59	ALT34440.1	Succinate-semialdehyde dehydrogenase	−2.326	0.5509	4.222	4834	0.001081	0.20
A0A9N7G3U9	ALT36182.1	NAD(P)H quinone oxidoreductase, PIG3 family	−2.227	0.5509	4.043	4834	0.00219	0.21
A0A9N7AN74	ALT36099.1	Triacylglycerol lipase	−2.161	0.5509	3.923	4834	0.003403	0.22
Q6A5I5	ALT36248.1	Citrate synthase	−2.156	0.5509	3.914	4834	0.003437	0.22
Q6AAQ4	ALT34727.1	Zinc-binding dehydrogenase	−2.145	0.5509	3.893	4834	0.003636	0.23
A0A9Q4C152	ALT36123.1	Oxidoreductase, aldo/keto reductase family protein	−2.12	0.5509	3.848	4834	0.004135	0.23
A0A9N7AP39	ALT34844.1	Uncharacterized protein	−2.09	0.5509	3.794	4834	0.004641	0.23
Q6A5L5	ALT36226.1	Zinc-type alcohol dehydrogenase-like protein	−2.085	0.5509	3.784	4834	0.004714	0.24
Q6A790	ALT35738.1	ABC transporter	−2.074	0.5509	3.765	4834	0.004975	0.24
Q6ABB7	ALT34536.1	Aminoglycoside phosphotransferase domain-containing protein	−2.046	0.5509	3.714	4834	0.005953	0.24
A0A9N7AY85	ALT36455.1	Trehalose 6-phosphate phosphatase	−2.04	0.5509	3.702	4834	0.006001	0.24
A0A9N7ALH2	ALW21_11825	D-alanine--D-alanine ligase	−2.038	0.5509	3.7	4834	0.006001	0.24
Q6A8U7	ALT35262.1	Tryptophanase	−1.997	0.5509	3.626	4834	0.00785	0.25
Q6A5D7	ALT36288.1	Conserved L-arabinose operon protein, hydrolase	−1.994	0.5509	3.621	4834	0.00785	0.25
Q6ABU7	ALT34429.1	Glycosyl hydrolase	−1.979	0.5509	3.592	4834	0.008577	0.25
Q6A5D9	ALT36286.1	L-arabinose utilization protein, glycerol dehydrogenase	−1.966	0.5509	3.568	4834	0.009209	0.26

Continued on next page

Accession #	Accession # on STRING	Protein name	Difference	SE of difference	t ratio	df	q value	Fold change
Q6A850	ALT35472.1	RecBCD enzyme subunit RecB	−2.354	0.6159	3.822	4834	0.004249	0.26
Q6ABL2	ALW21_00020	DNA replication and repair protein RecF	−1.955	0.5509	3.549	4834	0.009714	0.26
Q6A5V1	ALT36621.1	Peptide ABC transporter, ATP-binding protein	−2.583	0.6747	3.829	4834	0.004243	0.35
Q6A7V7	ALT35548.1	Biotin synthase	3.695	0.6159	6	4834	<0.000001	3.60
Q6A7C5	ALT35709.1	Conserved phage-associated protein	2.441	0.6159	3.963	4834	0.002971	5.44
Q6A8V6	ALT35254.1	Amino acid permease / DNA binding protein	3.433	0.5509	6.231	4834	<0.000001	10.80

Table S2. Proteomic changes *in C. acnes* in 5 nM epineprhine biofilms in comparison with in 5 nM epineprhine planktonic cultures. Yellow labeling means the proteins which were affected in control samples. Red color indicates downregulated proteins. Green color indicates upregulated proteins.

Accession #	Accession # on STRING	Protein name	Difference	SE of difference	t ratio	df	q value	Fold change
A0A085B0S7	ALT34593.1	Ribose 5-phosphate isomerase	−4.419	0.5347	8.265	4702	<0.000001	0.01
Q6A5D5	ALT36290.1	Anaerobic ribonucleoside-triphosphate reductase	3.553	0.8021	4.43	4702	0.000831	0.05
Q6A945	ALT35173.1	ATP-dependent dethiobiotin synthetase BioD	−4.156	0.5347	7.773	4702	<0.000001	0.06
A0A9Q4C1B0	ALT36376.1	Metallophosphoesterase	−3.064	0.5978	5.125	4702	0.000033	0.08
A0A828UFF1	AOH45217.1	Aminooxidase	−2.836	0.5347	5.304	4702	0.000015	0.09
A0A9N7AVS8	ALW21_06360	Ribulokinase	−3.555	0.5978	5.947	4702	<0.000001	0.11
A0A9Q4GER7	ALT35781.1	SPFH/Band 7/PHB domain protein	2.472	0.6549	3.775	4702	0.008047	0.11
Q6A5Z8	ALT36102.1	cAMP factor 3	−2.749	0.5978	4.598	4702	0.000405	0.11
A0A828UK58	ALT36285.1	Myo-inositol catabolism protein	−3.175	0.5978	5.311	4702	0.000015	0.11
A0A9N7G0V0	ALT34913.1	Tat pathway signal sequence domain protein	−2.152	0.5347	4.025	4702	0.003744	0.11
Q6AAK0	ALT34771.1	AP endonuclease, family 2	−3.058	0.5347	5.719	4702	0.000002	0.12
A0A9Q4BYX8	ALT35629.1	ABC transporter, solute-binding protein	−2.355	0.5347	4.404	4702	0.000879	0.12
A0A828SG38	ALT35236.1	ADP/GDP-polyphosphate phosphotransferase	−2.844	0.5978	4.757	4702	0.000201	0.14
A0A9Q4BXX9	ALT36531.1	Amidinotransferase	−3.166	0.5347	5.92	4702	<0.000001	0.20

Continued on next page

Accession #	Accession # on STRING	Protein name	Difference	SE of difference	t ratio	df	q value	Fold change
Q6ABE0	ALT34515.1	LUD domain-containing protein	−2.275	0.5347	4.255	4702	0.001528	0.21
A0A9Q4GGK6	ALT35136.1	30S ribosomal protein S13	−2.061	0.5347	3.854	4702	0.006339	0.22
A0A371N860	AOG29170.1	Succinate CoA transferase	−2.011	0.5347	3.761	4702	0.008188	0.22
A0A9N7FZU1	ALT34959.1	Oxoglutarate dehydrogenase (Succinyl-transferring), E1 componen	−3.237	0.5347	6.054	4702	<0.000001	0.22
Q6AAL0	ALT34763.1	Transaldolase	−2.09	0.5347	3.909	4702	0.005781	0.23
Q6ABE2	ALT34513.1	Cysteine-rich domain protein	−2.083	0.5347	3.896	4702	0.005825	0.24
Q6A8U7	ALT35262.1	Tryptophanase	−2.076	0.5347	3.883	4702	0.005876	0.24
A0A9Q4GEX3	ALT35364.1	Glycosyltransferase family 2 protein	−3.155	0.5978	5.278	4702	0.000016	0.24
Q6AAK3	ALT34770.1	Methylmalonate-semialdehyde dehydrogenase (Acylating)	−2.055	0.5347	3.844	4702	0.006346	0.24
A0A085B1W8	ALT36064.1	Inorganic pyrophosphatase	−4.837	0.7562	6.396	4702	<0.000001	0.25
A0A9Q4GGS6	ALT36130.1	ATP-dependent DNA helicase, RecQ family	−2.19	0.5347	4.096	4702	0.002908	3.00
A0A9Q4GEK0	ALT35999.1	Oxidoreductase, NAD-binding domain protein	−5.095	0.7562	6.737	4702	<0.000001	4.24
Q6A7U4	ALT35559.1	ACR, COG1399	2.312	0.5347	4.324	4702	0.00119	4.97

Continued on next page

AIMS Press

© 2024 the Author(s), licensee AIMS Press. This is an open access article distributed under the terms of the Creative Commons Attribution License (http://creativecommons.org/licenses/by/4.0)
